# Characteristics, Outcomes and Factors for Place of Death in Patients Admitted to Community-Based Palliative Care Services in Shanghai China: A Multicenter Retrospective Cohort Study

**DOI:** 10.1089/pmr.2024.0033

**Published:** 2024-10-23

**Authors:** Yanxia Lin, Chuchu Yan, Dongliang Yang, Murong Zhang, Haiying Gao, Anqi Xie, Jinwen Chang, Yiwen Mao, Yongxing Shi

**Affiliations:** ^1^ School of Nursing, Shanghai University of Traditional Chinese Medicine, Shanghai, China.; ^2^ Public Curriculum Teaching Department, Cangzhou Medical College, Cangzhou, China.; ^3^ Hospice Care Unit, Yingyuan Hospital, Shanghai, China.; ^4^ Hospice Care Unit, Jinshanwei Community Health Service Center, Shanghai, China.; ^5^ Hospice Care Unit, Zhongshan Community Health Service Center, Shanghai, China.; ^6^ Hospice Care Unit, Linfen Community Health Service Center, Shanghai, China.; ^7^ Hospice Care Unit, Changzheng Community Health Service Center, Shanghai, China.; ^8^ Shanghai Hospice Care Management Center, Shanghai, China.

**Keywords:** cohort studies, community health services, demography, end of life care, hospice care, palliative care, place of death

## Abstract

**Background:**

Community-based palliative care (CBPC) is only available in large cities in mainland China and little is known about who utilizes it.

**Objectives:**

This study examined the characteristics, outcomes, and factors associated with place of death (PoD) among inpatient CBPC patients.

**Design:**

This was a multicenter retrospective cohort study.

**Settings/Subjects:**

All patients admitted to the inpatient CBPC unit in four community health centers in 2021 in Shanghai, China, were included.

**Methods:**

Characteristics and outcome data were extracted from electronic health records and paper version notes between September 4 and December 29, 2022. PoD was followed up on May 12, 2023. Data were analyzed using descriptive analysis and categorized using two-step clustering. Decision tree analysis was used to identify factors associated with PoD.

**Results:**

The cohort admitted in 2021 included 290 participants (Age: 75.7 ± 12.7 years; Male: *n* = 155, 53.4%) including two children, with a mortality rate of 59.0% and a median length of stay (LoS) of 14 days upon December 29, 2022. The primary diagnosis for 80.3% of participants was tumor. Two clusters were identified. Cluster 1 was smaller than Cluster 2 (*n* = 45, 15.5% vs. *n* = 245, 84.5%) and dominated by noncancer participants (*n* = 37, 82.2%), whereas Cluster 2 included 91.8% (*n* = 225) tumor patients. Greatest significant differences in age, sex, marital status, education level, awareness of diagnosis and/or prognosis, mortality, LoS, and costs were found between the clusters. In total, 265 deaths derived from the cohort upon May 12, 2023, occur in inpatient CBPC units (75.5%), at home (18.9%), and in hospital wards (5.7%), influenced largely by participants’ marital status and age.

**Conclusions:**

Establishing contextualized inpatient CBPC services in more places nationwide that are tailored to different characteristics between cancer patients (i.e., younger and shorter inpatient stay) and noncancer patients (i.e., older and longer stay) is essential to maintain that more dying patients remain in their community.

## Key Message

A multicenter retrospective cohort study of 290 participants from four inpatient CBPC services in China revealed that the majority of patients were old, suffered from cancer, were conscious, and clinically stable at admission but utilized CBPC for a short period of time and two thirds died in CBPC unit. The cancer patients had significant differences from noncancer participants in terms of demographics, clinical conditions, timing of receiving inpatient CBPC, and outcomes indicating different trajectories.

### What is already known about the topic?

• Although the majority of the Chinese population die at home, CBPC is limited to several large cities in mainland China.
• Systematic reviews have shown that patients receiving CBPC are more likely to die at home and have fewer hospitalizations and emergency visits, shorter hospital stays, improved quality of life, and lower health care costs.

### What this paper adds?

• This multicenter retrospective cohort study identified that most inpatient CBPC users were elderly, largely diagnosed with cancer, and received CBPC for a very short period of time before death.
• The patient-level complexity of CBPC in China also determined by the cancer subgroup differed from the noncancer subgroup in many demographic and clinical aspects requiring specific care guidance tailored to the characteristics.
• Influenced by patients’ marital status and age, inpatient CBPC services have provided patients with an option for PoD, which might change the PoD distribution in different settings or care models following illness trajectories.

### Implications for practice, theory, and policy

• Improvement priorities for researchers, practitioners, and policymakers include reducing the inequality of access to CBPC, particularly for nonadult and noncancer patients.
• Specific clinical guidance for different subgroups utilizing CBPC services is needed.

## Introduction

Community-based palliative care (CBPC) refers to the active holistic care of individuals with severe illness, especially those near the end of life,^1^ offered at a community health center (CHC).^2,3^ CBPC at the end of life and terminal stages is beneficial for dying patients in the community. Systematic review evidence shows that CBPC programs increase the likelihood of seriously ill patients having their place of death (PoD) at home, fewer hospitalizations and emergency room visits, decreased hospital length of stay (LoS), improved quality of life, and lower health care costs.^4,5^ Given that most people prefer to remain in the community or at home, CBPC services can achieve significant coverage and reduce access inequality of services for patients with chronic, life-limiting health problems,^2,3,6^ and bring death back into the community.^7^ CBPC programs are being increasingly implementing worldwide,^4^ including mainland China.

Although palliative care is in an advanced stage of integration into the health system, particularly in hospitals in mainland China,^8^ CBPC services for end of life care (EoLC) have only been formally developed in large cities. Funded by the government since 2012,^9^ Shanghai has developed a model of CBPC that includes inpatient and outpatient care at one of 249 CHCs across 16 administrated regions as well as home-based care provided by the CHC-based medical team. CBPC services in Shanghai are embedded in settings at a CHC primarily commissioned for public health and primary care. Patients can transition between outpatient, inpatient, and home service based on the following criteria: patients scoring <70 on the Karnofsky Performance Scale (KPS) and an expected survival period of no more than 6 months according to the score of Palliative Performance Scale were eligible for home care, whereas patients scoring <50 on the KPS and an expected survival period of no more than 3 months were eligible for inpatient CBPC.^10^ In the current model, the inpatient services dominate while home care for dying patients is rare which is a common pattern issue across mainland China. Each CHC has one 10-bed unit providing inpatient palliative care including symptom management, comfort care, psychological support, and humanistic care through a multidisciplinary team comprising general practitioners (GPs), district nurses, social workers, psychologists, pharmacists, physiotherapists, traditional Chinese medicine doctors, nursing support workers, and volunteers.^11–13^ However, evidence shows that CBPC practices in Shanghai remain underutilized and underdeveloped. For example, bed utilization was as low as 48%,^14^ which could have been worsened by the COVID-19 pandemic.^15^ Less developed primary care with limited resources compared to secondary and tertiary care in China impacts those who use CBPC.^16^

Understanding the characteristics of patients utilizing CBPC services is valuable for improving and informing current practices.^17^ Specifically, patients’ personal and demographic factors (i.e., age, sex), illness-related factors (i.e., primary disease), and environmental factors (i.e., health care services) inform their PoD.^18–22^ Evidence concerning CBPC practices from China can also contribute to the international base because comparisons between countries and health care systems reveal how services and interventions work for individual patients and help identify the best solutions for their complex needs and problems.^23^

This study aimed to examine the characteristics, outcomes, and factors of PoD in patients admitted to four CBPC services receiving inpatient care at a CHC in Shanghai, China.

## Methods

### Study design and settings

A retrospective cohort study using a standardized chart review approach was conducted on patients admitted to the inpatient CBPC unit of four CHCs across four geographical areas in Shanghai, China. The RECORD checklist was used to guide this study.^24^ The study protocol can be accessed upon request.

Supplementary Appendix SA1 provides the information on the four CHCs. The settings were selected by considering the best practices in both the central and remote regions of Shanghai. A CBPC team typically consists of one doctor director, one head nurse, GP doctors and nurses, one social worker, and other members from different disciplines.

### Participants

All patients admitted to the inpatient CBPC unit in the four CHCs in Shanghai, China, from January 1 to December 31, 2021, were included. The sample size was estimated using the principle of ten multiples per variable.^25^ This study examined 22 variables; thus, a sample of at least 220 records was deemed necessary. One entire year was included to reduce sampling bias on admission differences in particular periods of a year. For example, some patients avoid admitting to any health care services during Ching Ming festival in April, Zhong Yuan Festival in July, and Winter Solstice in December, when it involves worship related to death. A preknowledge about the number of admissions each year in the selected CBPC units was obtained from the unit head nurse or director.

### Study procedures and data sources

Participants were identified through the electronic health record (EHR) system by limiting the timeframe of admission and verified by comparing them with handwritten admission/discharge records. A standardized chart was developed and used to extract data from various record sources (see Supplementary Appendix SA2). The following data were retrieved from the EHR: a) patient demographics (age, sex, marital status, and ethnicity); b) disease information (primary diagnosis, treatment history); c) clinical conditions at admission (consciousness, vital signs); d) admission details (admission method); e) outcomes (mortality); and f) service usage (LoS, cost, and payment). Data on participants’ sociodemographic characteristics (religion, educational level, and occupation) and awareness of diagnosis and/or prognosis were extracted from the paper version of the CBPC notes (separate from the EHR system). All data were collected between September 4 and December 29, 2022. The PoDs of those discharged were collected on May 12, 2023, from follow-up notes routinely completed by nurses. All data were extracted by one author and checked against the records for accuracy by another author, including the links between different sources against identity number, name, and bed number. Any disagreements were resolved through discussion with a third investigator. A data collection protocol was developed and the research team was trained prior to data extraction.

### Variables

Data on participants’ characteristics and outcomes were collected retrospectively. Sociodemographic data, including age, sex, marital status, educational level, occupation, ethnicity, and religion, were collected by clinicians at admission. The clinical characteristic data included primary diagnosis, awareness of diagnosis and/or prognosis, surgery history, chemotherapy, radiotherapy, method of admission, consciousness, and vital signs at admission. The outcome data included mortality, PoD, LoS, costs, and payment. Mortality rate referred to the patients who died in inpatient CBPC units, while all participants died or were discharged upon December 29, 2022. Awareness of diagnosis and/add or prognosis referred to whether the patient was aware of the illness threatening life and the terminal or end of life stage and this was assessed by clinicians using a checklist via conversation with patients and/or their family members at admission. The method of admission was categorized as walking with or without assistance, sitting in a wheelchair, or lying on a stretcher. Vital signs included respiratory rate (RR), body temperature (BT), heart rate (HR), and blood pressure (BP). PoD referred to the location where the patient died after utilizing CBPC upon May 12, 2023.

### Statistical analysis

All analyses were performed using SPSS 27.0 software (IBM Corporation, NY). Descriptive statistics were used to analyze participants’ characteristics and outcomes. If the data were normally distributed, continuous variables were presented as means with standard deviations (SDs), whereas categorical variables were analyzed using raw numbers and frequencies or percentages (%). Non-normally distributed data were presented as medians with interquartile ranges (IQRs). A two-step cluster analysis was conducted to categorize participants based on their characteristics and outcomes. Differences between clusters obtained from the two-step cluster analysis were analyzed using t-tests and chi-square tests. *p* ≤ 0.05 was considered statistically significant.

The classification and regression tree approach was used in the decision tree analysis to evaluate the factors that influenced participants’ PoD. Age, sex, occupation, education level, marital status, religion, ethnicity, tumor/nontumor status, consciousness at admission, awareness of diagnosis and/or prognosis, and survival time following admission were added to the model as independent variables.

Missing values were replaced with the mean and mode for continuous variables and categorical variables, respectively, which is a single-imputation approach.^26^ Restricting the analysis to patients with a complete dataset may yield different (and poorly generalizable) inferences.^27^

### Ethics approval

The Shanghai Ethics Committee for Clinical Research approved this study (Reference: SECCR/2022-111-01). As CHCs in Shanghai lack institutional review boards, the authors obtained permission to conduct this study from the director at each institution, who provided signed consent. The requirement for informed consent was waived. All data were de-identified to protect cohort members’ privacy.

## Results

**FIG. 1.**
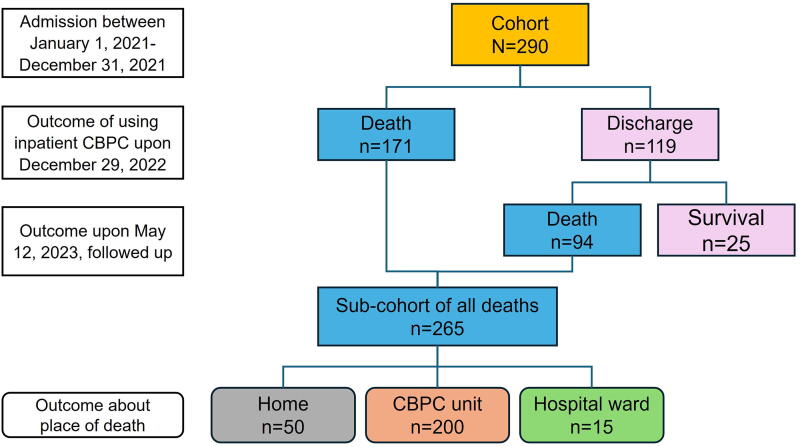
The cohort flow chart. The cohort included all patients who admitted to the four inpatient CBPC units at CHCs between January 1, and December 31, 2021 (*n* = 290). The clinical outcomes comprised death (*n* = 171) and discharge (*n* = 119), which were evaluated upon December 29, 2022. Those discharged were followed up routinely by CBPC nurses and another 94 deaths were added to the analysis of place of death (PoD) upon May 12, 2023. A subgroup of all deaths derived from the cohort summed up to 265, as laid at the bottom.

The final cohort comprised 290 participants (Fig. 1). No records were excluded from the study. Supplementary Appendix SA3 shows the number of participants from each of the four investigated CHCs and the percentages of missing variables.

### Participants’ characteristics

The 290 participants were, on average, 75.7 years old (SD = 12.7, range = 8–101), including two nonadult male patients, aged 8 and 10 years, respectively. Approximately 53.4% (*n* = 155) of the 290 sample were male (Table 1). The majority of participants were married (*n* = 242, 83.4%), retired (*n* = 261, 90.0%), and had no religion (*n* = 277, 95.5%). All participants were of Han ethnicity, and 57.9% (*n* = 168) reported their highest level of education was middle school. Among the 290 participants, 80.3% (*n* = 233) had a primary diagnosis of tumor. The five most common types of cancer were lung (*n* = 65, 27.9%), colorectal (*n* = 23, 10.0%), pancreatic (*n* = 21, 9.0%), stomach (*n* = 19, 8.2%), and liver (*n* = 17, 8.3%). Participants’ nontumor conditions were mainly cardiovascular (*n* = 23, 40.4%) and cerebrovascular diseases (*n* = 16, 28.1%). Among those with tumors (*n* = 233), 58.4% had a history of surgery and 40.3% had received chemotherapy before admission to inpatient CBPC. Most were conscious at admission (*n* = 256, 88.3%), used a stretcher or wheelchair (*n* = 258, 89.0%), and were aware of their diagnosis and/or prognosis (*n* = 213, 73.4%). Most participants’ vital signs at admission were normal, but a minority had an abnormal BP (*n* = 81, 27.9%), HR (*n* = 57, 19.6%), RR (*n* = 44, 15.2%), or BT (*n* = 27, 9.3%). The mortality rate for all participants during admission upon December 29, 2022 was 59.0% (*n* = 171), and the median LoS of all participants in the CBPC group was 14 days (IQR = 5–58.25). The median total cost per participant was 4,976.3 Chinese yuan (IQR = 2356.94–14420.49), which is equivalent to a median daily cost of 323.9 Chinese yuan. Approximately 96.2% of participants paid CBPC costs under health care insurance.

**Table 1.** Characteristics of Participants Admitted to the Four Inpatient CBPC Services (*n* = 290)

Variable	Total (*n* = 290)	Cluster 1 (*n* = 45)	Cluster2 (*n* = 245)	*t*/ c^2^	*p*
Age (years), mean (SD), range	75.7 (12.7), 8–101	87.7 (7.8)	73.5 (12.2)	10.145	<0.001
Age level (years), *n* (%)					
≤17	2 (0.7)	0 (0)	2 (0.8)		
18–64	34 (11.7)	0 (0)	34 (13.9)	13.044	0.001
≥65	254 (87.6)	45 (100.0)	209 (85.3)		
Sex, *n* (%)					
Male	155 (53.4)	5 (11.1)	150 (61.2)	38.372	<0.001
Female	135 (46.6)	40 (88.9)	95 (38.8)		
Marital status, *n* (%)					
Married	242 (83.4)	43 (95.6)	197 (80.4)	6.113	0.013
Unmarried^a^	48 (16.6)	2 (0.4)	48 (1.6)		
Education level, *n* (%)					
Primary school and below	98 (33.8)	32 (71.1)	66 (26.9)	33.297	<0.001
Middle school	168 (57.9)	12 (26.7)	156 (63.7)		
College and above	24 (8.3)	1 (2.2)	23 (9.4)		
Occupation, *n* (%)					
Retired	261 (90.0)	45 (100.0)	216 (88.2)	—	0.049
Employed	19 (6.6)	0 (0)	19 (7.8)		
Unemployed/ Farmer	10 (3.4)	0 (0)	10 (4.0)		
Ethnicity, *n* (%)					
Han	290 (100.0)	45 (100.0)	245 (100.0)	—	—
Religion, *n* (%)					
Yes	13 (4.5)	2 (4.4)	11 (4.5)	0.000	1.000
No	277 (95.5)	43 (95.6)	234 (95.5)		
Primary diagnosis at admission, *n* (%)					
Tumor	233 (80.3)	8 (17.8)	225 (91.8)	132.039	<0.001
Nontumor	57 (19.7)	37 (82.2)	20 (8.2)		
Surgery history^b^, *n* (%)					
Yes	136 (58.4)	3 (37.5)	133 (59.1)	0.729	0.393
No	97 (41.6)	5 (62.5)	92 (40.9)		
Chemotherapy history^b^, *n* (%)					
Yes	94 (40.3)	0 (0)	94 (41.8)	4.001	0.045
No	139 (59.7)	8 (100.0)	131 (58.2)		
Radiotherapy history^b^, *n* (%)					
Yes	31 (13.3)	0 (0)	31 (13.8)	0.357	0.550
No	202 (86.7)	8 (100.0)	194 (86.2)		
Consciousness at admission, *n* (%)					
Conscious	256 (88.3)	42 (93.3)	214 (87.3)	1.316	0.251
Unconscious	34 (11.7)	3 (6.7)	31 (12.7)		
Awareness of diagnosis and/or prognosis, *n* (%)					
Aware	213 (73.4)	19 (42.2)	194 (79.2)	26.632	<0.001
Unaware	77 (26.6)	26 (57.8)	51 (20.8)		
Way of admission, *n* (%)					
Walking in	32 (11.0)	0 (0)	32 (13.1)	11.010	0.004
On wheelchair	78 (26.9)	8 (17.8)	70 (28.6)		
On stretcher	180 (62.1)	37 (82.2)	143 (58.3)		
RR (n/min), mean (SD)	19.5 (1.54)	18.8 (1.3)	19.6 (1.6)	−3.485	<0.001
RR level (n/min), *n* (%)					
<16	2 (0.7)	1 (2.2)	1 (0.4)	4.251	0.119
16–20	246 (84.8)	41 (91.1)	205 (83.7)		
>20	42 (14.5)	3 (6.7)	39 (15.9)		
BT (°C), mean (SD)	36.7 (0.53)	36.7 (0.4)	36.7 (0.6)	−0.193	0.847
BT level (°C), *n* (%)					
<37.3	263 (90.7)	42 (93.3)	221 (90.2)	3.094	0.377
37.3–38.0	18 (6.2)	3 (6.7)	15 (6.1)		
38.1–39.0	7 (2.4)	0 (0)	7 (2.9)		
>39.0	2 (0.7)	0 (0)	2 (0.8)		
HR (n/min), mean (SD)	87.3 (14.67)	80.9 (7.2)	88.5 (15.4)	−5.262	<0.001
HR level (n/min), *n* (%)					
<60	5 (1.7)	0 (0)	5 (2.0)	14.637	<0.001
60–100	233 (80.4)	44 (97.8)	189 (77.2)		
>100^c^	52 (17.9))	1 (2.2)	51 (20.8)		
BP (mmHg), *n* (%)					
SBP<90 or DBP<60	34 (11.7)	0 (0)	34 (13.9)	14.869	<0.001
SBP 90–140 and DBP 60–90^c^	209 (72.1)	43 (95.6)	166 (67.7)		
SBP ≥140 or DBP ≥90	47 (16.2)	2 (0.4)	45 (18.4)		
Mortality, *n* (%)				6.171	0.013
Discharge	119 (41.0)	26 (57.8)	93 (37.9)		
death	171 (59.0)	19 (42.2)	152 (62.1)		
LoS (days), median (IQR)	14 (5, 59.5)	156 (46, 244)	12 (4.5, 27)	−7.325	<0.001
Total cost (Chinese yuan), median (IQR)	4976.3 (2356.9, 14420.5)	37627.5 (12091.9, 63815.2)	4093.4 (2077.9, 9175.9)	−7.410	<0.001
Daily cost (Chinese yuan), median (IQR)	323.9 (228.0, 506.5)	255.9 (222.2, 310.1)	347.6 (230.4, 524.8)	−3.225	0.001
Payment approach, *n* (%)					
Health insurance	279 (96.2)	45 (100.0)	234 (95.5)	1.050	0.306
Out-of-pocket payment	11 (3.8)	0 (0)	11 (4.5)		

^a^ single, widowed, or devoiced.

^b^ only for tumor participants: Total *n* = 233, Cluster 1 *n* = 8, Cluster 2 *n* = 225.

^c^ including two children.

RR, respiratory rate at admission; BT, body temperature; HR, heart rate; BP, blood pressure at admission; SBP, systolic blood pressure; DBP, diastolic blood pressure; LoS, length of stay; IQR, interquartile range.

### Participants categories

The two-step cluster analysis generated two clusters of participants with different characteristics (Table 1). The size of Cluster 1, which was dominated by noncancer participants (*n* = 37, 82.2%), was much smaller than that of Cluster 2 (*n* = 45, 15.5% vs. *n* = 245, 84.5%), in which most participants had a primary diagnosis of cancer (*n* = 225, 91.8%). Compared to those in Cluster 2, participants in Cluster 1 were older (87.7 vs. 73.5 years), more likely to be female than male (88.9% vs. 38.8%), more likely to be married than unmarried (single, widowed, or divorced) (95.6% vs. 80.4%), and had a lower education level (26.9% vs. 71.1% of primary school and below). In Cluster 1, 100% were retired, while in Cluster 2 88.2% were employed or unemployed. Compared to Cluster 2, Cluster 1 had no history of chemotherapy (100% vs. 41.8%). Moreover, they were more likely to not be aware of diagnosis and/or prognosis (57.8% vs. 20.8%), have a normal HR (97.8% vs. 77.2%) and BP (95.6% vs. 67.7%), be on stretcher or wheelchair at admission rather than walking in (100.0% vs. 86.9%), be discharged rather than die in CBPC units (57.8% vs. 37.9%). They also had a much longer LoS (156 vs. 12 days) and a higher total cost (37,627.5 vs. 4,093.4 Chinese yuan) but a lower daily cost (255.9 vs. 347.6 Chinese yuan).

### Place of death and its factors

**FIG. 2.**
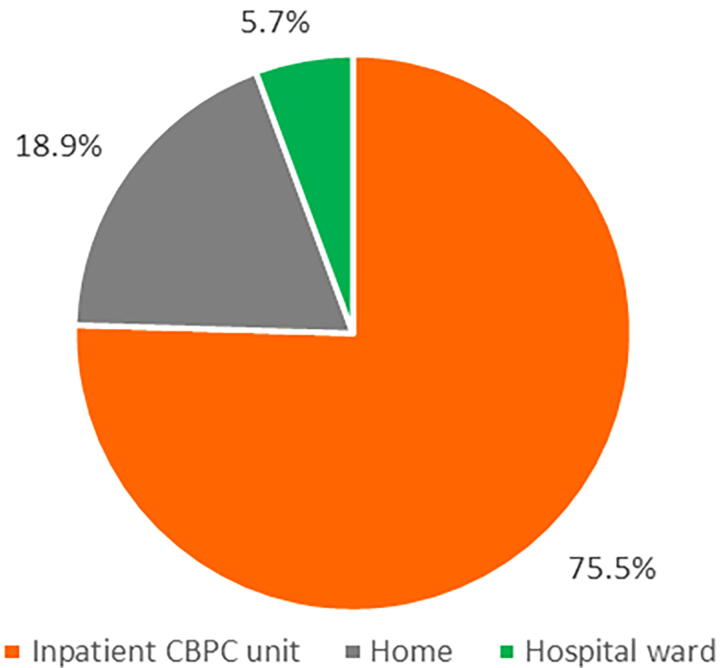
The PoD after using inpatient CBPC services (*n* = 265). A subgroup of 265 deceased participants died in CBPC units (*n* = 200, 75.5%), home (*n* = 50, 18.9%), and hospital wards (*n* = 15, 5.7%) after utilizing inpatient CBPC services.

Upon May 12, 2023, a subgroup of 265 deaths were derived from the cohort, including those who died during inpatient CBPC (*n* = 171) and those who died after discharge during follow-up by May 12, 2023 (*n* = 94) (Fig. 1). As shown in Figure 2, the most common PoD in the subgroup was inpatient CBPC units (*n* = 200, 75.5%), followed by home (*n* = 50, 18.9%), and hospital wards (*n* = 15, 5.7%). The 265 deceased participants’ characteristics are presented in Supplementary Appendix SA4.

**FIG. 3.**
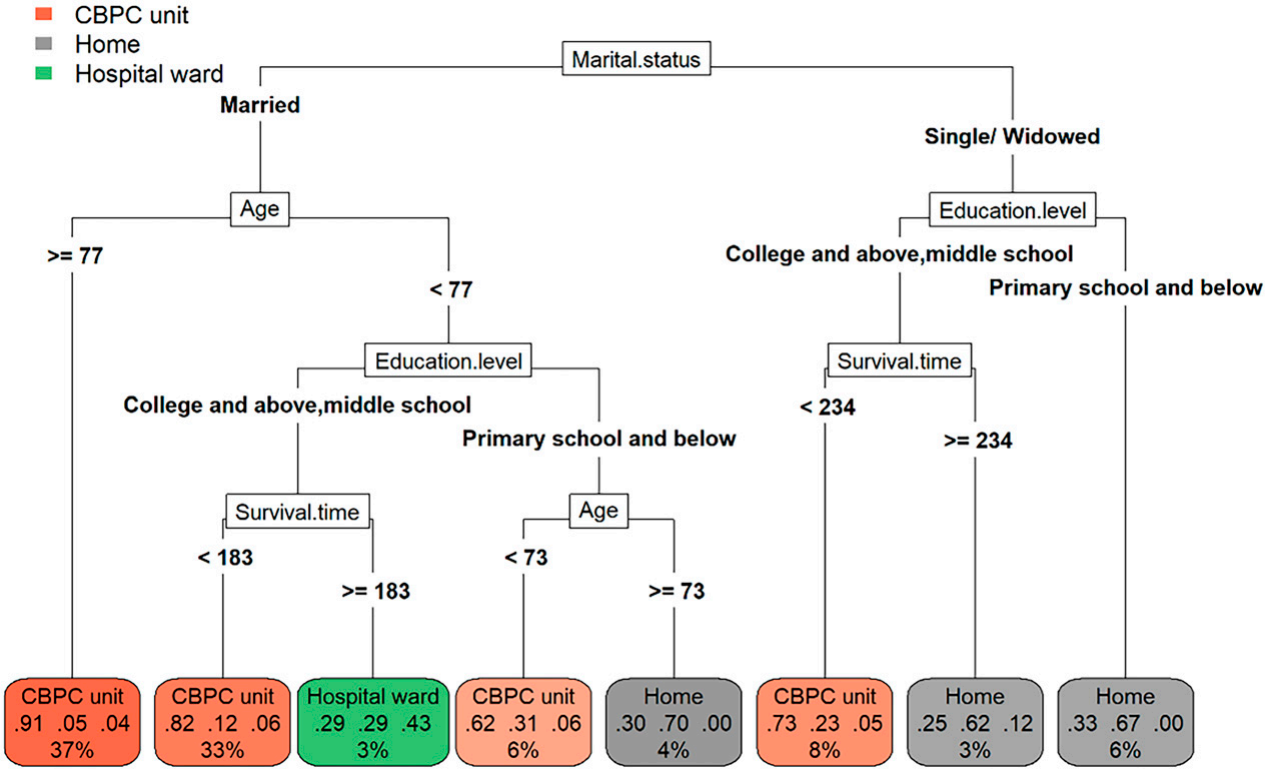
The decision tree of factors for PoD (*n* = 265). The decision tree flow diagram shows the factors shaping the participants’ choice of PoD including: marital status, education level, age, and survival time post admission. The tree’s root is on the top marked as the factor marital status in white box and its leaves are boxes in different colors at the bottom illustrating the three places where participants died, with different factors connected by lines in between forming the branches of tree. From up bottom, the higher the factor box is the more influential the factor is on the decision of PoD. A most likely PoD can be identified following a continuous line from top to the bottom box. Deaths in inpatient CBPC unit, at home, and in hospital ward are presented in red, gray, and green coloured boxes, respectively. The darker color suggests a larger statistical effect size and this is further quantified by the numbers in each box. For example, the first box on the left includes 37% of the 265 deaths, and within which 91% occur in inpatient CBPC unit, 5% at home, and 4% in hospital ward. Therefore, this box shows the PoD in inpatient CBPC unit. The most important results are shown in the first two boxes from the left side.

Factors influencing participants’ choice of PoD included marital status, age, education level, and survival time after admission to inpatient CBPC services (Fig. 3). A key finding is shown in the first two red boxes from the left side of Figure 3 that participants who were married and aged ≥77 years or who were married and aged <77 years but had a higher education level (middle school and above) and a survival time shorter than 183 days were more likely to die in inpatient CBPC units rather than at home or in hospital wards.

## Discussion

This retrospective cohort study aimed to identify the characteristics, outcomes, and factors associated with PoD in patients who utilized four inpatient CBPC services at CHCs in Shanghai, China. The majority of the population were elderly, suffered from cancer, were conscious, and clinically stable at admission but utilized inpatient CBPC for a short period of time and more than half died in CBPC unit. Most participants were cancer patients with significant differences from noncancer participants in terms of demographics, clinical conditions, timing of receiving inpatient CBPC and outcomes indicating different trajectories. Influenced by patient-level factors, inpatient CBPC services have provided patients with an option for PoD because the majority of them die in the setting after admission. The availability of inpatient CBPC services might change the PoD from a hospital-dominated model for a particular illness, or from a home-dominated pattern in more general population across China^21,28^ to a more even distribution in different settings or care models that is similar to the situation in many other countries.^29–31^ The patient-level characteristics identified in our study also suggest inequality in access to CBPC in different subgroups, which indicates the need for further interventions and practice improvement.

Our first suggestion is to increase CBPC coverage for nonadults because their access to CBPC was extremely limited in our study; only two dying children were admitted to the four inpatient CBPC services within one year. There is the potential to include more dying children in CBPC services because within all children’s death approximately 16.1%– 41. 57% occur at home each year in Shanghai.^32,33^ CBPC for nonadults may be more complex than that for adults due to the barriers to pediatric palliative care, such as the lack of a trained workforce and resources in primary care.^34,35^ By addressing these barriers, nonadults can benefit from CBPC; for example, to be maintained in the community and close to their parents.

Second, our findings show that CBPC services should expand their coverage to noncancer patients because most patients currently using inpatient CBPC services in Shanghai, China had cancer. Data from the official website show that although tumors were a common cause of death in Shanghai in 2021, circulatory and respiratory diseases also frequently resulted in mortality.^36^ Despite the continuously growing number of patients with nonmalignant diseases using EoLC internationally, compared to cancer patients, the proportion of patients with noncancer illnesses is extremely low.^37–40^ A systematic review of evidence found that palliative care provides benefits for patients with noncancer illness.^41^ Thus, more efforts are needed to increase noncancer patients’ access to CBPC in mainland China.

In line with the increasing access to CBPC, our findings further suggest the development of different CBPC guidelines for cancer and noncancer patients because the two groups differ significantly regarding demographic and clinical characteristics and outcomes. In our study, noncancer participants were much older, more likely to be discharged rather than die in inpatient CBPC but had a relatively longer stay and higher total costs than cancer participants. These varied user characteristics indicate different levels of complexity in CBPC provision.^42^ The existing evidence indicates that cancer and noncancer patients have different trajectories, with different symptoms and palliative care needs.^43–47^ Given that the quantity and quality of evidence for symptom treatment among cancer patients are far better than those for noncancer patients, additional efforts are needed to develop CBPC guidelines for noncancer patients based on the evidence generated in our study and others.

We further recommend developing CBPC practice guidelines that differ from other palliative care models in mainland China by considering participants’ characteristics. Compared with those receiving hospital-based palliative care, patients using inpatient CBPC in our study were older (77.5 vs. 52.2–59.8 years)^48,49^ and had different types of cancer.^48^ At least five clinical models of palliative care are being delivered across care settings worldwide, including outpatient clinics, inpatient consultation teams, acute palliative care units, CBPC, and hospice care.^50–52^ Each model serves a different patient population.^53^ Patients with the highest level of suffering and complexity may choose hospital-based acute palliative care, whereas home-based service is more appropriate for patients with poor performance status and a low-to-moderate symptom burden.^52^ The CBPC model in Shanghai under investigation has similarities with the international CBPC model but also has uniqueness in terms of being embedded in primary care and providing three different modes of palliative care by one team. Therefore, there is an urgent need to develop evidence-based guidelines for CPBC practices in mainland China.

### Strength and limitations

To the best of our knowledge, this is the first multicenter cohort study to investigate the characteristics, outcomes, and factors of PoD among inpatient CBPC patients in mainland China, where EoLC at the community level is limited to a small number of large cities. This study systematically describes the population characteristics and identifies two categories of users. These findings provide the first portrait of CBPC complexity at the patient level, forming a part of the evidence base for further studies and clinical practice changes. Our study also makes an important contribution to the international evidence base by offering insights into the cultural and social factors influencing access to CBPC in China. However, this study has several limitations. First, there is a selection bias owing to convenience sampling of only four CHCs, which negatively impacts the representativeness of the data to the referent large population. Although the proportion of missing data is small, it might reduce the representativeness of the selected sample and cause bias, as well as the efficiency and validity of the conducted analyses, further reducing the generalizability of the findings.

## Conclusion

CBPC services change the pattern of death by providing people with a place of care and death within their communities. By revealing the patient-level complexity of CBPC practices in Shanghai, China, our findings support the value and feasibility of establishing contextualized CBPC services in more places nationwide that are tailored to different characteristics between the cancer patients and noncancer population. To encourage more CBPC services, the priorities for further research and practice include reducing inequality in access to CBPC, expanding the coverage of CBPC for nonadults and noncancer illnesses, and developing specific practice guidance for these subgroups. Further studies are needed to examine the symptoms of patients with CBPC and to develop and evaluate effective context-sensitive CBPC programs.

## Data Sharing Statement

De-identified data supporting the findings of this study are available for ethically approved studies from the corresponding author upon reasonable request. A data-transfer agreement is required.

## Supplementary Material

Supplementary Data S1

Supplementary Appendix SA1

Supplementary Appendix SA2

Supplementary Appendix SA3

Supplementary Appendix SA4

## Abbreviations Used

BP: blood pressure
BT: body temperature
CBPC: community-based palliative care
CHC: community health center
COVID-19: Coronavirus Disease 2019
DBP: diastolic blood pressure
EHR: electronic health record
EoLC: end of life care
GP: general practitioner
HR: heart rate
IQRs: interquartile ranges
KPS: Karnofsky Performance Scale
LoS: length of stay
NY: New York
PoD: place of death
RECORD: The REporting of studies Conducted using Observational Routinely collected health Data
RR: respiratory rate
SBP: systolic blood pressure
SDs: standard deviations

## References

[1] Radbruch L, De Lima L, Knaul F, et al. Redefining palliative care—a new consensus-based definition. J Pain Symptom Manage 2020;60(4):754–764; doi: 10.1016/j.jpainsymman.2020.04.027 32387576 PMC8096724

[2] WHO. Planning and Implementing Palliative Care Services: A Guide for Programme Managers. 2016. Available from: https://www.who.int/publications/i/item/planning-and-implementing-palliative-care-services-a-guide-for-programme-managers [Last accessed: 7 July, 2023].

[3] WHO. Integrating Palliative Care and Symptom Relief into Primary Health Care: A WHO Guide for Planners, Implementers and Managers. 2018. Available from: https://www.who.int/publications/i/item/integrating-palliative-care-and-symptom-relief-into-primary-health-care [Last accessed: 12 October, 2023].

[4] Vernon E, Hughes MC, Kowalczyk M. Measuring effectiveness in community-based palliative care programs: A systematic review. Soc Sci Med 2022;296:114731; doi: 10.1016/j.socscimed.2022.114731 35131612

[5] NICE. Chapter 14 Community Palliative Care: Emergency and Acute Medical Care in Over 16s: Service Delivery and Organisation. 2018. Available from: https://www.nice.org.uk/guidance/ng94/evidence/14.community-palliative-care-pdf-172397464601 [Last accessed: 5 August, 2023].

[6] Mills J, Abel J, Kellehear A, et al. Access to palliative care: The primacy of public health partnerships and community participation. Lancet Public Health 2021;6(11):e791–e792; doi: 10.1016/S2468-2667(21)00213-9 34634238

[7] Sallnow L, Smith R, Ahmedzai SH, et al. Lancet Commission on the Value of Death. Report of the lancet commission on the value of death: Bringing death back into life. Lancet 2022;399(10327):837–884; doi: 10.1016/S0140-6736(21)02314-X 35114146 PMC8803389

[8] Clark D, Baur N, Clelland D, et al. Mapping levels of palliative care development in 198 Countries: The Situation in 2017. J Pain Symptom Manage 2020;59(4):794–807.e794; doi: 10.1016/j.jpainsymman.2019.11.009 31760142 PMC7105817

[9] Shanghai Health Bureau. Shanghai Hospice Care Services Initiative. 2012. Available from: http://wsjkw.sh.gov.cn/xxfb4/20180525/28098.html [Last accessed: 6 December, 2019].

[10] Shanghai MHC. Shanghai Palliative Care Service Regulation. 2020. Available from: https://wsjkw.sh.gov.cn/jcws2/20200812/4653c9a4830b46e08b883f01fa5e0aab.html [Last accessed: 5 October, 2021].

[11] Wu Y, Feng D, Shi Y, et al. Practice and thinking of hospice care in community healthcare center. Chinese Nursing Manag 2019;19:811–815.

[12] Yu J, Jin J, Zhang M, et al. Multi-disciplinary coordination model in palliative care based on integrated medicine. Chinese Journal of General Practitioners 2023;22:654–656.

[13] Wang C, Cheng W, Cao Y. Research on the supply of palliative care in Shanghai. Chinese Primary Health Care 2020;34:34–36.

[14] Jing L, Liu H, Liu K, et al. Evaluation of the palliative care input and output effect of community health service centers in Shanghai. Chinese General Practice 2016;19:4178–4182.

[15] Wang C, Wang Z, Wang G, et al. COVID-19 in early 2021: Current status and looking forward. Signal Transduct Target Ther 2021;6(1):114; doi: 10.1038/s41392-021-00527-1 33686059 PMC7938042

[16] Li X, Lu J, Hu S, et al. The primary health-care system in China. The Lancet 2017;390(10112):2584–2594. 10.1016/S0140-6736(17)33109-4 29231837

[17] Rose PM. Patients’ characteristics informing practice: Improving individualized nursing care in the radiation oncology setting. Support Care Cancer 2018;26(10):3609–3618; doi: 10.1007/s00520-018-4210-5 29728842

[18] Gomes B, Higginson IJ. Factors influencing death at home in terminally ill patients with cancer: Systematic review. BMJ 2006;332(7540):515–521. 16467346 10.1136/bmj.38740.614954.55 PMC1388126

[19] Cohen J, Pivodic L, Miccinesi G, et al. International study of the place of death of people with cancer: A population-level comparison of 14 countries across 4 continents using death certificate data. Br J Cancer 2015;113(9):1397–1404; doi: 10.1038/bjc.2015.312 26325102 PMC4815784

[20] Lin Y, Long-Sutehall T, Myall M. Transferring home to die from critical care units: A scoping review of international practices. J Crit Care 2021;65:205–215; doi: 10.1016/j.jcrc.2021.06.012 34243069

[21] Wang W, Liu Y, Ye P, et al. Trends and associated factors in place of death among individuals with cardiovascular disease in China, 2008-2020: A Population-Based Study. The Lancet Regional Health - Western Pacific 2022;21:100383; doi: 10.1016/j.lanwpc.2022.100383 35540560 PMC9079349

[22] Murtagh FEM, Bausewein C, Petkova H, et al. Understanding Place of Death for Patients with Non Malignant Conditions: A Systematic Literature Review. 2012. Available from: http://www.netscc.ac.uk/hsdr/files/project/SDO_FR_08-1813-257_V01.pdf [Last accessed: 23 December, 2016].

[23] Higginson IJ. End-of-life care: Lessons from other nations. Journal of Palliative Medicine 2005;8 (Suppl 1):S161–S173; doi: 10.1089/jpm.2005.8.s-161 16499464

[24] Benchimol EI, Smeeth L, Guttmann A, et al. RECORD Working Committee. The REporting of studies conducted using Observational Routinely-collected health Data (RECORD) statement. PLoS Med 2015;12(10):e1001885; doi: 10.1371/journal.pmed.1001885 26440803 PMC4595218

[25] Sackett DL, Haynes RB, Guyatt GH, et al. Clinical Epidemiology: A Basic Science for Clinical Medicine. Little, Brown & Company: Boston, MA; 1991.

[26] Emmanuel T, Maupong T, Mpoeleng D, et al. A survey on missing data in machine learning. J Big Data 2021;8(1):140; doi: 10.1186/s40537-021-00516-9 34722113 PMC8549433

[27] MIT Critical Data. Secondary Analysis of Electronic Health Records. Springer: Cham (CH); 2016. 31314219

[28] Weng L, Hu Y, Sun Z, et al. Place of death and phenomenon of going home to die in Chinese adults: A Prospective Cohort Study. Lancet Reg Health West Pac 2021;18:100301; doi: 10.1016/j.lanwpc.2021.100301 35024647 PMC8671632

[29] Pivodic L, Pardon K, Morin L, et al. Place of death in the population dying from diseases indicative of palliative care need: A cross-national population-level study in 14 countries. J Epidemiol Community Health 2016;70(1):17–24; doi: 10.1136/jech-2014-205365 26202254

[30] Cross SH, Warraich HJ. Changes in the place of death in the United States. N Engl J Med 2019;381(24):2369–2370; doi: 10.1056/NEJMc1911892 31826345

[31] Public Health England. Palliative and end of life care profiles. 2023. Available from: https://fingertips.phe.org.uk/profile/end-of-life/data#page/0/gid/1938132883/pat/46/par/E12000004/ati/165 [Last accessed: 19 October, 2023].

[32] Xu L, Zhang L, Sun E, et al. Survey of deaths in children under the age of 5 years in Pudong New Area Shanghai between 2011-2017. Maternal and Child Health Care of China 2019;34:4772–4774.

[33] Tang X, Xue H. Analysis of the causes of death in children under the age of 5 years in Jinshan District Shanghai between 2009-2018. Maternal and Child Health Care of China 2019;34:5328–5331.

[34] Cheng L, Liu W, Yu L, et al. Influencing factors of pediatric hospice care practices perceived by professional caregivers: A Qualitative Study. Military Nursing 2023;40:11–14; doi: 10.26914/c.cnkihy.2022.030746

[35] Gao L, Xing J. Analysis of influencing factors and countermeasures of hospice care for cancer children. Chinese Medical Ethics 2021;34:995–998,1009.

[36] Shanghai MHC, Shanghai M. Shanghai Municipal Health Statistics in 2021. 2022. Available from: https://wsjkw.sh.gov.cn/tjsj2/20220704/a540b90305ae4c54bf870b3804c6f84c.html [Last accessed: 15 October, 2022].

[37] Amy G, Eleanor K, Steven Edward O, et al. Palliative care for non-cancer conditions in primary care: A time trend analysis in the UK (2009–2014). BMJ Supportive & Palliative Care 2020:bmjspcare-2019-001833; doi: 10.1136/bmjspcare-2019-001833 31932476

[38] Kingston AEH, Kirkland J, Hadjimichalis A. Palliative care in non-malignant disease. Medicine 2020;48(1):37–42; doi: 10.1016/j.mpmed.2019.10.010

[39] Romanò M, Oldani S, Reina V, et al. Palliative care for patients with end-stage, non-oncologic diseases-a retrospective study in three public palliative care Departments in Northern Italy. Healthcare 2022;10(6):1031; doi: 10.3390/healthcare10061031 35742082 PMC9222892

[40] Lin L-S, Huang L-H, Chang Y-C, et al. Trend analysis of palliative care consultation service for terminally ill non-cancer patients in Taiwan: A 9-year Observational Study. BMC Palliat Care 2021;20(1):181; doi: 10.1186/s12904-021-00879-z 34823512 PMC8614035

[41] Quinn KL, Shurrab M, Gitau K, et al. Association of receipt of palliative care interventions with health care use, quality of life, and symptom burden among adults with chronic noncancer illness: A systematic review and meta-analysis. JAMA 2020;324(14):1439–1450; doi: 10.1001/jama.2020.14205 33048152 PMC8094426

[42] Carrasco-Zafra MI, Gómez-García R, Ocaña-Riola R, et al. Level of palliative care complexity in advanced cancer patients: A multinomial logistic analysis. J Clin Med 2020;9(6); doi: 10.3390/jcm9061960 PMC7356562 32585859

[43] Bandeali S, Des Ordons AR, Sinnarajah A. Comparing the physical, psychological, social, and spiritual needs of patients with non-cancer and cancer diagnoses in a tertiary palliative care setting. Pall Supp Care 2020;18(5):513–518; doi: 10.1017/S1478951519001020 31771668

[44] Lai WS, Liu IT, Tsai JH, et al. Hospice delivery models and survival differences in the terminally ill: A Large Cohort Study. BMJ Support Palliat Care 2021;0:1–10; doi: 10.1136/bmjspcare-2021-003262 34916240

[45] Harrison KL, Kotwal AA, Smith AK. Palliative care for patients with noncancer illnesses. JAMA 2020;324(14):1404–1405; doi: 10.1001/jama.2020.15075 33048142 PMC7771527

[46] Murray S, McLoughlin P. Illness trajectories and palliative care: Implications for holistic service provision for all in the last year of life. In: International Perspectives on Public Health and Palliative Care. (Sallnow L, Kumar S and Kellehear A. eds.) Routledge: London; 2013; pp. 30–51.

[47] RACGP. RACGP aged care clinical guide (Silver Book): Part A. Palliative and End-Of-Life Care. 2022. Available from: https://www.racgp.org.au/getattachment/55aea74c-fbe9-4d01-8cbc-425948225cb8/Palliative-care.aspx [Last accessed: 3 June, 2023].

[48] Zhang H, Shang L, Huang Z. Practice of palliative care for hospitalized patients with advanced cancer. Chinese Nursing Management 2016;16:407–410.

[49] Zhu J. The effect of palliative care intervention combining hope theory on the quality of life amon patients with advanced tumours. Electronic Journal Of Practical Clinical Nursing Science 2020;5:44.

[50] Chung H, Harding R, Guo P. Palliative care in the greater China Region: A systematic review of needs, models, and outcomes. J Pain Symptom Manage 2021;61(3):585–612; doi: 10.1016/j.jpainsymman.2020.08.040 32916261

[51] Brereton L, Clark J, Ingleton C, et al. What do we know about different models of providing palliative care? Findings from a systematic review of reviews. Palliat Med 2017;31(9):781–797; doi: 10.1177/0269216317701890 28376681

[52] Hui D, Bruera E. Models of palliative care delivery for patients with cancer. J Clin Oncol 2020;38(9):852–865; doi: 10.1200/jco.18.02123 32023157 PMC7082156

[53] Comans T, Nguyen KH, Stafford-Bell F, et al. Cost comparison of different models of palliative care delivery. Australas J Ageing 2021;40(1):90–93; doi: 10.1111/ajag.12843 32965056

