# Supplementary material for: Characteristics, Outcomes and Factors for Place of Death in Patients Admitted to Community-Based Palliative Care Services in Shanghai China: A Multicenter Retrospective Cohort Study
Source: Palliat Med Rep. 2024 Oct 23;5(1):481–91. doi: 10.1089/pmr.2024.0033 (PMC11512087; doi:10.1089/pmr.2024.0033)
Supplement: Supplementary Appendix SA1 [file pmr.2024.0033_supp_datasa1.docx]

**Appendix I** The four inpatient CBPC at CHC under study

| Name of CHC and location | Beds of palliative and hospice ward | Region of Shanghai | Time of palliative and hospice service initiation | Number of patients offered home palliative care in 2021 | Specialized Team members | MDT |
| --- | --- | --- | --- | --- | --- | --- |
| Xincheng Road CHC, Jiading District | 10 | Remote region | 2012 | 2 | 17: four doctors, 16 nurses, one social worker | Including the following members: TCM (Traditional Chinese Medicine) doctor, occupational therapist, psychotherapist, pharmacist, nutritionist, and a group of nursing support workers, and volunteers. |
| Jinshan county CHC, Jinshan District | 10 | Remote region | 2012 | 0 | 17: 8 doctors, 8 nurses, one social worker |  |
| Linfeng Road CHC, Jingan District | 26 | Central region | 1993 | 0 | 17: 8 doctors, 8 nurses, one social worker |  |
| Zhongshan Road CHC, Songjiang District | 12 | Remote region | 2012 | 1 | 18: 3 doctors, nurses, one social worker |  |
