# Supplementary figures and images for: Characteristics, Outcomes and Factors for Place of Death in Patients Admitted to Community-Based Palliative Care Services in Shanghai China: A Multicenter Retrospective Cohort Study
Source: Palliat Med Rep. 2024 Oct 23;5(1):481–91. doi: 10.1089/pmr.2024.0033 (PMC11512087; doi:10.1089/pmr.2024.0033)

**
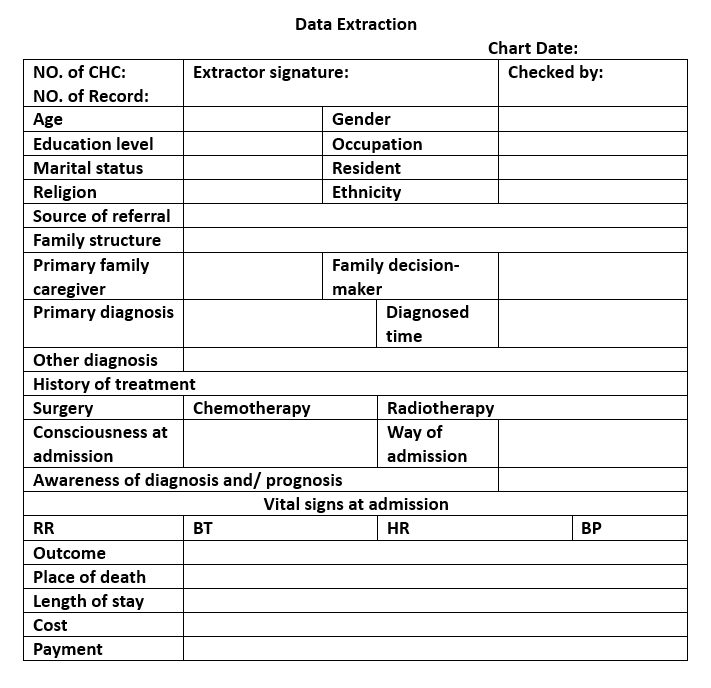
Appendix II** Data extraction chart

Supplement: Supplementary Appendix SA2 [file pmr.2024.0033_supp_datasa2.docx]
