# Supplementary material for: Characteristics, Outcomes and Factors for Place of Death in Patients Admitted to Community-Based Palliative Care Services in Shanghai China: A Multicenter Retrospective Cohort Study
Source: Palliat Med Rep. 2024 Oct 23;5(1):481–91. doi: 10.1089/pmr.2024.0033 (PMC11512087; doi:10.1089/pmr.2024.0033)
Supplement: Supplementary Appendix SA3 [file pmr.2024.0033_supp_datasa3.docx]

**Appendix III** The percentages of missing values

| Variable | % missing values in CHC 1 | % missing values in CHC 2 | % missing values in CHC 3 | % missing values in CHC 4 | % missing values in the four CHCs |
| --- | --- | --- | --- | --- | --- |
| Number of records identified (N) | 40 | 65 | 133 | 52 | 290 |
| Religion (%) | 15 | 3.1 | 0.8 | 94.2 | 20.0 |
| Education level (%) | 7.5 | 4.6 | 2.3 | 92.3 | 19.7 |
| Awareness of diagnosis and/or prognosis (%) | 2.5 | 3.1 | 6.0 | 1.9 | 4.1 |
| Occupation (%) | 0 | 3.1 | 0 | 0 | 0.7 |
| Marital status (%) | 0 | 3.1 | 0 | 0 | 0.7 |
