# Supplementary material for: Characteristics, Outcomes and Factors for Place of Death in Patients Admitted to Community-Based Palliative Care Services in Shanghai China: A Multicenter Retrospective Cohort Study
Source: Palliat Med Rep. 2024 Oct 23;5(1):481–91. doi: 10.1089/pmr.2024.0033 (PMC11512087; doi:10.1089/pmr.2024.0033)
Supplement: Supplementary Appendix SA4 [file pmr.2024.0033_supp_datasa4.docx]

**Appendix IV** Characteristics of the deceased participants upon May 12, 2023 (n=265)

| Variable | N (%)/ Median (IQR) |
| --- | --- |
| Age (years), mean (SD) | 75.6 (12.9) |
| Age level, n (%) |  |
| ≤17 | 2 (0.8) |
| 18 - 64 | 31 (11.7) |
| ≥65 | 232 (87.5) |
| Gender, n (%) |  |
| Male | 142 (53.6) |
| Female | 123 (46.4) |
| Marital status |  |
| Married | 220 (83.0) |
| Unmarried | 45 (17.0) |
| Education level, n (%) |  |
| Primary school and below | 87 (32.8) |
| Middle school | 154 (58.1) |
| College and above | 24 (9.1) |
| Occupation, n (%) |  |
| Retired | 239 (90.2) |
| Employed | 16 (6.0) |
| Unemployed/ Farmer | 10 (3.8) |
| Ethnicity, n (%) |  |
| Han | 265 (100.0) |
| Religion, n (%) |  |
| Yes | 13 (4.9) |
| No | 252 (95.1) |
| Primary diagnosis at admission, n (%) |  |
| Tumour | 221 (83.4) |
| Non-tumour | 44 (16.6) |
| Consciousness, n (%) |  |
| Conscious | 233 (87.9) |
| Unconscious | 32 (12.1) |
| Awareness of diagnosis and/or prognosis, n (%) |  |
| Aware | 196 (74.0) |
| Unaware | 69 (26.0) |
| Length of stay, median (IQR) | 13 (5, 35) |
| Total cost, median (IQR) | 4730.58 (2297.835, 11670.56) |
| Place of death, n (%) |  |
| CBPC unit | 200 (75.5) |
| Home | 50 (18.9) |
| Hospital ward | 15 (5.7) |
